# Survivorship care and support following treatment for breast cancer: a multi-ethnic comparative qualitative study of women’s experiences

**DOI:** 10.1186/s12913-016-1625-x

**Published:** 2016-08-18

**Authors:** Charlotte Tompkins, Karen Scanlon, Emma Scott, Emma Ream, Seeromanie Harding, Jo Armes

**Affiliations:** 1 Breast Cancer Care, 5-13 Great Suffolk Street, London, SE1 0NS UK; 2 King’s College London, Florence Nightingale Faculty of Nursing & Midwifery, James Clerk Maxwell Building, 57 Waterloo Road, London, SE1 8WA UK; 3 School of Health Sciences, Faculty of Health & Medical Sciences, University of Surrey, Duke of Kent Building, Guildford, GU2 7XH UK; 4 King’s College London, Diabetes & Nutritional Sciences Division, Franklin-Wilkins Building, 150 Stamford Street, London, SE1 9NH UK

**Keywords:** Breast cancer, Multi-ethnic, Survivorship care, Follow-up, Aftercare, Qualitative

## Abstract

**Background:**

As the number of breast cancer survivors continues to rise, Western populations become more ethnically and socially diverse and healthcare resources become ever-more stretched, follow-up that focuses on monitoring for recurrence is no longer viable. New models of survivorship care need to ensure they support self-management and are culturally appropriate across diverse populations. This study explored experiences and expectations of a multi-ethnic sample of women with breast cancer regarding post-treatment care, in order to understand potential barriers to receiving care and inform new models of survivorship care.

**Methods:**

A phenomenological qualitative research design was employed. In-depth interviews were conducted with women from diverse socio-demographic backgrounds in England, who completed treatment for breast cancer in the 12 months prior to the study. Data were analysed using Framework Analysis.

**Results:**

Sixty-six women participated and reported expectations and needs were unmet at follow-up. Whilst there were more commonalities in experiences, discernible differences, particularly by ethnicity and age, were identified relating to three key themes: emotional responses on transition to follow-up; challenges communicating with healthcare professionals at follow-up; and challenges finding and accessing information and support services to address unmet needs.

**Conclusions:**

There are cultural differences in the way healthcare professionals and women communicate, not necessarily differences in their post-treatment needs. We do not know if new models of care meet survivors’ needs, or if they are appropriate for everyone. Further testing and potential cultural and linguistic adaptation of models of care is necessary to ensure their appropriateness and acceptability to survivors from different backgrounds. New ways of providing survivorship care mean survivors will need to be better prepared for the post-treatment period and the role they will have to play in managing their symptoms and care.

**Electronic supplementary material:**

The online version of this article (doi:10.1186/s12913-016-1625-x) contains supplementary material, which is available to authorized users.

## Background

### Breast cancer survival

The number of people surviving cancer is increasing. Worldwide, the number of cancer survivors within five years of diagnosis was estimated to be 32.6 million in 2012, up from 28.7 million in 2008 [1]. Whilst breast cancer is the most common cancer in women, with 1.67 million new cases diagnosed worldwide in 2012 (25 % of all cancers) [1], it also has one of the highest survival rates [2].

Whilst the number of breast cancer survivors in England is increasing and is expected to double from two million in 2010 to four million by 2030 [3], the socio-demographic profile of these survivors is also changing. Western populations are ageing and becoming more diverse in terms of their ethnic and socioeconomic composition [4]. For example, the White ethnic group is the largest ethnic group in England and Wales but has decreased over the last two decades, whilst minority ethnic groups have more than doubled in size [5, 6] (See Table 1). The Asian/Asian British ethnic groups saw some of the largest increases between the 2001 and 2011 Censuses [5] and the African ethnic group has grown faster than any other minority group over the past two decades [6].

**Table 1:** Ethnic groups in England and Wales (2011)

Ethnic group	England & Wales (%)
White (White British)	86.0 (80.5)
Mixed/Multiple Ethnic Groups	2.2
Asian/Asian British	7.5
Black/African/Caribbean/Black British	3.3
Other	1.0

(Source: Office for National Statistics, 2012)

Although breast cancer incidence and survival rates vary across ethnic groups, breast cancer remains the most common cancer among all ethnic groups in the UK and rates among the largest ethnic groups (South Asian, Black Caribbean and Black African) are falling more in line with those of the largely White population, due in part to their ageing populations and adoption of more Westernised lifestyles [7]. Most worrying is the poorer cancer outcomes of women from these ethnic groups, which has largely been attributed to later presentation with disease compared with the main White population [8]. Later stage presentation may reflect more aggressive disease, lack of awareness about symptoms, mistrust of the healthcare system, lower uptake of screening or delays in diagnosis [8]. Breast cancer incidence is lower among socioeconomically disadvantaged women compared with women in a higher socioeconomic position (SEP), but survival is poorer [9–11]. Explanations for this include later stage presentation and lower uptake of breast cancer screening, lifestyle choices, co-morbidities and poorer access to the best treatments, information and support [12, 13].

### Experience of breast cancer post-treatment

Although survival rates are improving, breast cancer survivors may experience physical, social and psychological issues following treatment [14–19]. Fear of recurrence is one of the most pressing concerns [15, 20–26], often triggered by physical symptoms, and can lead to psychological distress, depression and anxiety [27, 28]. Fatigue, loss of energy and lymphoedema are commonly cited physical symptoms [8–12] as are hormonal changes and menopausal symptoms [20, 22, 24]. Alongside changes in sexual function, including decreased libido [22–25], changes in physical appearance and body image concerns [20, 22–25] can lead to intimacy and relationship issues [22–25]. Coming to terms with loss and a sense of apprehension about the future are also key concerns. Women can feel abandoned and vulnerable once treatment ends, separated from the perceived safety and security of hospital and healthcare professionals (HCPs) [14, 15, 18, 29–31]. Loss of opportunities and life roles have also been highlighted as issues [15, 20, 21, 24]. Once treatment has finished, women strive to resume family, work and social responsibilities [20]. However, this comes at a time when they may have reduced social support, not just from HCPs but also family and friends who believe life returns to normal once treatment is over. As a result, some women experience difficulties returning to normal life, as they feel permanently changed by the experience of breast cancer [32, 33]. It could be suggested women in a lower SEP and women from ethnic minority backgrounds may experience more emotional, social and functional problems post-treatment, as a consequence of undergoing more aggressive treatments to manage the disease. However, there is a paucity of research exploring potential ethnic and social variations in women’s needs and experiences post-treatment [34].

### Changing models of survivorship care

As more people survive cancer, there is a growing need for Western healthcare systems to consider new approaches to the delivery of survivorship care for their increasingly diverse populations. However, improving the quality of care for women with breast cancer as they move beyond treatment, in a climate of increasingly limited healthcare resources, presents new challenges in public health.

There is a move internationally from a ‘one-size fits all’ approach to follow-up (focusing on clinical monitoring to detect recurrence) to more personalised ‘aftercare’ [29]. Research showed that clinical follow-up tended to overlook patients physical, emotional and social concerns, despite evidence that timely referral to support services improves quality of life [31]. This has led to wide acceptance among HCPs and health policy makers that the current aftercare system does not meet patients’ needs [16]. Survivorship care in the UK now includes survivors and their primary care providers being given a treatment summary and survivorship care plan, which outline treatments received, the risk of late effects of treatment and follow-up care needs; the aim being to improve communication and coordinate care between hospital oncology services, primary care and cancer survivors [29]. Those at low risk of recurrence are encouraged to self-manage, with support from HCPs, i.e. take greater responsibility for their own health, wellbeing and care. The extent to which these models of care are being implemented varies worldwide due to the differing healthcare delivery systems in place. In addition, little research has considered the impact of these new approaches on minority ethnic groups and women in a lower SEP.

### Aims of the study

As survivorship care is being redesigned, it is important to explore the experiences of women from different social and ethnic backgrounds to ensure new models of care are culturally appropriate to meet their needs. This paper aimed to compare the experiences and expectations of a sample of White, Black and South Asian women of varying SEP with breast cancer across major cities in England, who were receiving traditional follow-up care (focused on clinical monitoring to detect recurrence), in order to better understand potential barriers to these groups receiving care and identify any gaps in care, so as to inform new models of care being implemented.

## Methods

### Research design

A qualitative research design was employed. In-depth interviews were conducted with women from diverse ethnic and social backgrounds who completed treatment for primary breast cancer in the previous 12 months.

### Recruitment and sample

Participants were recruited from eight hospitals in England (North England (*n* = 3), Inner London (*n* = 3) and South East England (*n* = 2). Hospitals were chosen as they serve large ethnic minority populations and the availability of regular follow-up clinics in which potential participants could be recruited. During recruitment all hospitals followed a traditional model of post-treatment follow-up (hospital-based appointments with doctors, held at regular, pre-determined intervals). Women were eligible to participate if they had completed hospital treatment for primary invasive breast cancer within the previous 12 months, were >30 years and English-speaking. Women were ineligible if they were unable to speak and understand English.

Between October 2011 and August 2012 over 200 eligible women were introduced to the study by either their consultant, breast care nurse, research nurse or the researcher (KS or CT) when they attended follow up appointments. Women were asked for written consent to be contacted by a member of the research team one week later, allowing them time to read the study participant information document, before deciding whether to participate in the study or not. This involved seeking the women’s permission to record their name, address and phone number. Of the 151 patients who expressed an interest in taking part in the study and provided consent to contact, a total of 110 were able to be contacted and completed screening questionnaires. Women were informed that the study aimed to recruit a diverse sample (using quota-sampling) of women to enable us represent potential differences in views and experiences that women may have post-treatment. The screening questionnaire included providing socio-demographic information on age, ethnicity, ability to speak English and SEP and clinical information regarding treatment received and completion dates). Of those, 11 were found to be ineligible (e.g. beyond 12 months post treatment or unable to speak English) and one refused to participate in the study. All women were informed that they would be provided with a £10 store voucher payment for participating in the study.

### Procedure

A non-proportional quota sample was chosen from women who expressed an interest in taking part and were eligible to participate. In order to gain maximum variation with regard to demographic characteristics, the sampling frame comprised the following subgroups: ethnicity (White British, Black African, Black Caribbean, Indian, Pakistani, Bangladeshi); age (30–50, 51–70, 71+ years); and SEP (using The National Statistics Socio-Economic Classification (NS-SEC) groupings: groups 1 and 2 combined, managerial and professional occupations, to reflect higher SEP and groups 3 to 5 combined, intermediate, supervisory or routine occupations, to reflect lower SEP), as well as type of breast cancer treatment (surgery, chemotherapy and radiotherapy) and time since treatment completion (within previous six months or within previous six to 12 months). Women were then selected to populate the various subgroups, with the aim of recruiting a minimum of 10 women to each subgroup. After providing written consent to be interviewed, interviews were conducted between November 2011 and February 2013, either at the participant’s home, a local cancer support service or in the hospital. All interviews were conducted using a topic guide and were digitally audio-recorded, with consent, and transcribed verbatim.

This paper is based on a larger study exploring the post-treatment physical, emotional, social and financial support needs of a multi-ethnic sample of women with breast cancer, within the first 12 months of treatment completion (see Additional file 1 for the full topic guide). The findings reported here stem from the analysis for this larger piece of research. Broad questions were asked about where women sought information and support to address their post-treatment needs. The types of questions were general in nature to allow full accounts of women’s experiences to be shared (see Table 2 for questions relating to follow-up). Follow-up was not the sole focus of the study but during the analysis it became clear that follow-up was an important area of unmet need for the women involved.

**Table 2:** Interview questions relating to follow-up

What information and support services have you used to address your needs since you finished breast cancer treatment, including those from NHS and other statutory service providers, and from other (non-statutory) providers? Who provided, when, and where? What do you think about the support that the hospital provided you about life after breast cancer treatment? What other support would you like to be available to patients following treatment to help them adjust/ cope with the physical and emotional impact? Who should deliver it, how and when?	

All interview transcripts were given unique ID numbers and did not include any participant identifiers, thus protecting participants’ anonymity.

### Analysis

Data were analysed using Framework Analysis [35–37]. There were three broad stages: i) data management, ii) development of descriptive accounts, and iii) development of explanatory accounts. Firstly, an analytical framework was developed based on a priori themes (reflected in interview question headings). This provided a matrix of rows (one for each participant) and columns (one for each subtheme). The matrices were grouped by ethnic background to facilitate comparison between ethnic groups. Interview transcripts were read, coded and indexed using the Framework facility in NVivo 9.2, where data were synthesised into the appropriate theme headings within the matrix. The summarised data were then examined, facilitated by the matrix structure, to explore individual and group similarities and differences by age, ethnicity, socioeconomic status and clinical characteristics. Coding was conducted by CT and checked on a randomised selection of transcripts for initial entry into the framework by a second researcher (KS) (*n* = 10). Further to this, KS & ES explored themes relating specifically to follow-up that had originally been identified by CT.

## Results

Sixty-six women were interviewed. Table 3 outlines their demographic characteristics. Women were between 34 and 84 years of age (mean 54 years). Of the 43 women from ethnic minority groups, 28 were born abroad and 15 were UK-born. The majority of women from ethnic minority groups in the lower SEP categories were born outside the UK and just over half of the ethnic minority women in the higher SEP categories were also born overseas. Table 4 provides clinical information. Most (*n* = 50) were interviewed within six months of treatment completion and had attended at least one hospital follow-up appointment (*n* = 48).

**Table 3:** Participants’ demographic characteristics

Ethnic Group		*N*
	White British	23
	Indian	7
	Pakistani	9
	Asian Other	1
	*Sub-total*	17
	Black Caribbean	14
	Black African	7
	*Sub-total*	21
	Other/Mixed ethnicity	5
Age		*N*
	30–50	27
	51–70	29
	≥71	10
Recruitment location		*N*
	North of England	26
	Inner London	21
	South-East	19
Socio-economic position (SEP)		*N*
NS-SEC 1 & 2 - Managerial, administrative and professional occupations - Intermediate occupations		35
NS-SEC 3, 4, 5 - Small employers and own account workers - Lower supervisory and technical occupations - Semi-routine and routine occupations		22
Never worked/long-term unemployed/other		9

**Table 4:** Participants’ clinical information

Months since end of active treatment	*N*
0–6	50
6–12	16
Number of follow-up appointments	*N*
0	8
≥1	48
Unknown	10

Participants, irrespective of ethnicity, SEP, age group, geographical location or time since treatment completion, reported their expectations and supportive care needs were unmet whilst attending hospital follow-up appointments. Whilst there were more commonalities in women’s experiences, through Framework Analysis, we identified discernible differences, particularly by ethnicity and age, relating to the following three key themes: emotional response on transition to follow-up; challenges communicating with HCPs at follow-up; and challenges finding and accessing information and support services to address unmet needs. This paper presents the commonalities identified across the sample but goes on to highlight where nuanced differences between socio-demographic groups exist. The commonalities and differences in expectations and experiences are summarised in Table 5 and elaborated further in the text below.

**Table 5:** Commonalities and differences in breast cancer patients’ follow-up experiences

Theme	Commonalities across groups	Differences by ethnicity	Differences by SEP	Differences by age
Emotional response on transition to follow-up	Abrupt end to treatment and appointments - feel abandoned, out of hospital ‘safety net’ Unprepared and uncertain about what to expect at the end of treatment and what follow-up care would entail	White British women shared the most about their feelings Black African women born overseas relieved frequency of appointments was reducing; did not feel abandoned	No discernible differences identified	No discernible differences identified
Challenges communicating with HCPs at follow-up	Lack of contact with Breast Care Nurse (BCN) - Surprised/disappointed BCNs not at follow-up appointments and/or do no make contact with women but: - Reluctance to contact BCNs Appointments focus on the physical; no opportunity to ‘talk’ particularly about emotional concerns Appointments rushed, impersonal so were not reassuring. Unable/uncomfortable asking questions, leading to unanswered questions Lack of continuity of care; unknown HCPs at follow-up appointments	White British women shared most about communication challenges; Black African women shared least Women born overseas reported language issues and needing someone with them at follow-up appointments; struggled to ask questions White British women reported HCPs manner changed – from personable and caring to rushed and unsympathetic Black African women did not mind seeing different HCPs at follow-up appointments	Indian & Pakistani women born overseas and in a lower SEP needed to be accompanied to appointments	Focusing on the physical was a positive for women aged 71+ as they were reassured cancer had gone
Challenges finding and accessing information and support services to address unmet needs	Reliance on written information post-treatment Women wanted someone to talk to: - expected BCN would fill this role - wanted to talk to other women who had breast cancer Would like telephone calls with BCN to discuss how they are feeling Ad-hoc signposting to information and support services - linked to availability of services - uptake of services more likely when signposted by trusted BCN	Language – women born overseas were given written information they could not read. Limited availability of culturally-specific information for Black African, Black Caribbean and South Asian women Black Caribbean and South Asian women wanted to *talk* to a BCN to get information; preferring verbal communication Women from minority ethnic groups wanted signposting to sources of information that considered culture and religion. Limited uptake of services by Black Caribbean and Black African women Black African women said peer support should be available but would not use (scared to/other commitments)	Indian & Pakistani women born abroad and in a lower SEP relied on family members to read information for them	Women under 50 reported a lack of signposting to information and support

### Emotional response on transition to follow-up

White British women shared the most about their feelings post-treatment. However, across ethnic groups (except Black African women), similar feelings were discussed. Women described an abrupt end to treatment; going from one extreme to another in terms of the contact and support they received from HCPs. Women felt ‘*abandoned’*, ‘*cast-adrift’* and out of the ‘*safety net’* of the hospital.

*All of a sudden, that's it, full stop. And it's like, you know, it's like being stranded in the middle of nowhere* (UK-born Pakistani, 30–50 years. 6–12 months post-treatment).

Black African women voiced a different response to ending treatment; rather than feeling ‘*abandoned’* they expressed relief that the burden of frequent treatment appointments was over, which conflicted with their work and social commitments. Follow-up appointments were also seen as anxiety-provoking by Black African women. One Black African woman said follow-up appointments made her worried as *‘you feel, “what are they going to say? What is the problem?”’* This woman also said she wanted to move on from cancer but it was difficult to do with so many appointments: ‘*you think, “when am I going to get my life back*?”’

Women did not know what to expect at the end of treatment or what the structure and content of follow-up care would be like. This resulted in them feeling unprepared for life after treatment. For example, some women felt unprepared for the ongoing nature of certain side effects:

*There is information about things like side effects and stuff like that but…you’d hear about the fatigue and you think “okay, as soon as you finish chemo, fatigue will go” but it didn’t, and it would have been helpful to be…told a bit more that, you know, some of the side effects are much…longer than you anticipate* (UK-born Mixed ethnicity, 30–50 years, 0–6 months post-treatment).

*There should be more things about preparing people for the ending and in terms of what to expect, what, I’m afraid not just what to expect… How do you adjust?* (UK-born Mixed ethnicity, 30–50 years. 6–12 months post-treatment).

### Challenges communicating with HCPs at follow-up

#### Disappointment with lack of breast care nurse (BCN) contact

White British women shared the most about their challenges communicating with HCPs, whilst Black African women shared very little. This appeared to be related to higher expectations among White British women for interactive dialogue and communication with their HCP at their follow-up appointment, compared to Black African women who were more likely to rely on their HCP to lead the dialogue. Many women expressed a desire for a continuation of the expert nursing skills they experienced during diagnosis and treatment. Women expressed surprise and disappointment that BCNs were not present at follow-up appointments. Indeed, the biggest complaint across groups was a lack of contact with a BCN, either there being less opportunity to talk to a BCN or not seeing them at all after treatment. Regardless of ethnicity, most women felt the BCN should be present at follow-up appointments. Despite wanting more contact with BCNs, there was reluctance to initiate contact with them; either because women felt BCNs would be busy with newly diagnosed patients, they would be wasting their time, or they had tried unsuccessfully to contact a BCN. However, in one locality, BCNs telephoned all women after treatment, which made women feel more able to contact their BCN if required.

*It’s just like you’re left, where are you going to go? I know there are the nurses I can go and talk to but still you can’t, you feel like you’re wasting your time if you’re ask every little thing and probably you’re wasting their time* (Born abroad Pakistani, 30–50 years. 0–6 months post-treatment).

*I think when I had follow-up appointment she [BCN] should have been there but she wasn’t… I didn’t get to talk to her at all. I didn’t even see her on that day, she wasn’t there* (Born abroad Indian, 51–70 years, 0–6 months post-treatment).

*‘[BCNs have been] really good… Even now she’ll ring me once a week to see how I’m doing and everything’* (UK-born Pakistani, 30–50 years, 0–6 months post-treatment).

#### Communication barriers

Women born overseas reported needing a relative with them at appointments to enable them to fully understand the context of conversations with HCPs. Indian and Pakistani women born overseas who needed to be accompanied to appointments tended to fall into the lower SEP category as well. They struggled to ask questions, in part, due to the hurried and impersonal nature of follow-up appointments, lack of continuity of HCPs and no supportive/facilitative BCN being present at follow-up. Language barriers were also discussed. Despite these women being able to speak and understand English, women born overseas worried about whether they would be understood when talking with an accent and felt less confident asking questions due to their limited English proficiency. Some women took English-speaking family members with them to interpret.

*I don’t understand if I don’t have one of my children next to me. I keep asking them. Always there has to be someone with me. She [daughter] took over because she kept asking the doctor because sometimes I can’t explain what I want to say* (Born abroad Other ethnicity, 51–70 years. 0–6 months post-treatment).

#### Focusing on the physical - little opportunity to discuss emotional concerns

Across ethnic groups, women mentioned appointments focused on the physical (e.g. wound healing, post-radiotherapy skin care, breast examination) and there was no opportunity to ‘talk’ about how they were feeling and coping after treatment. This focus on physical concerns meant there was insufficient time to comprehensively assess how well women were managing emotionally and socially after treatment.

*When you come to an appointment, they just deal with the breasts, they don’t ask me… They’re just dealing with the body, yes. But we need more people to deal with the person* (UK-born Black Caribbean, 30–50 years. 0–6 months post-treatment).

However, whilst this physical focus was generally perceived as negative, a minority of women welcomed it. Mixed and South Asian (Other) women born overseas and women over 70 years found appointments reassuring specifically because they focused on the physical; checking that the cancer had gone and they were recovering well. They did not appear to need the appointment for emotional support.

*They said every time, very good, your condition, so I am happy and come home* (Born abroad South Asian (Other), 51–70 years, 0–6 months post-treatment).

#### Hurried nature of appointments

Across ethnic groups, women reported their follow-up appointments were not long enough to discuss their post-treatment concerns (physical or emotional). Instead they felt appointments were ‘*rushed’* and *‘short and sweet*.’

*It was like two minutes and I’m out* (Born abroad Black Caribbean, 30–50 years. 6–12 months post-treatment).

Brief follow-up appointments contributed to those participants hoping for reassurance that they were disease-free feeling worried they were not being sufficiently examined to determine this. In addition, some Asian and Black women of varying SEP reported they did not receive enough information (either verbally or in writing) about being breast awareness to ensure they knew the signs of recurrence.

*You think, ‘are they feeling it properly?’ And then sometimes you do come out of there and think ‘oh, am I just another number or another one that they’ve seen?’* (White British, 30–50 years. 6–12 months post-treatment).

Women stated they often waited for follow-up appointments to raise issues with HCPs. However, in some cases, the hurried nature of appointments contributed to women feeling uncomfortable or unable to ask questions. Women reported they were left with unanswered questions about practical and emotional concerns, including returning to work and how to move on with their lives. Likewise, some women felt there was no one there to ask questions, for example, if the BCN was not available or did not attend follow-up appointments. The issue of unanswered questions was also linked to lack of continuity of HCPs. Some women also described how they felt they did not know *what* to ask, particularly if it was their first follow-up appointment, whilst a couple of White British women mentioned HCPs’ manner changed post-treatment; they were not as friendly or sympathetic. This may have lead to women feeling uncomfortable asking questions.

*Previous appointments have been more friendly… They’ve got to put you at ease more whereas afterwards when you’re well again perhaps they feel that doesn’t matter so much* (White British, 71+, 0–6 months post-treatment).

#### Lack of continuity of HCPs

Women across different ethnic groups expected follow-up appointments to be conducted with the same doctor/consultant and BCN involved in their treatment, believing this constituted the most suitable aftercare, as they knew their case. However, many participants were surprised and disappointed this did not happen. Only Black African women did not report feeling dissatisfied with seeing different HCPs at follow-up.

*You don’t necessarily see the same person do you? Which is a shame really. I know doctors say, ‘it doesn’t matter’ you know, ‘we’re generic,’ you know, ‘we’ve got all the information on the computer,’ but I think it’s not the same for a patient, a patient likes to see the same [doctor]* (White British, 71+ years. 0–6 months post-treatment).

Participants felt less at ease in follow-up appointments with unknown HCPs, questioning how well they knew their medical history. Appointments with unknown HCPs further deterred women from asking questions, as they did not have an established and trusted relationship.

*When somebody strange comes in, I don’t know that person and that person doesn’t know me* (Born abroad Black Caribbean, 30–50 years. 6–12 months post-treatment).

### Challenges finding and accessing information and support services to address unmet needs

All women were given written information in English irrespective of their spoken English skills. As well as proving difficult to understand, this information rarely considered religious or cultural influences on post-treatment recommendations, e.g. food preferences and dietary restrictions. Some Indian and Pakistani women born abroad, who were in a lower SEP, reported being unable to read English and relied on family members to read information provided - if it was read at all.

Additionally, there were differences in how participants from different minority ethnic groups preferred to receive information. Women overwhelming said they wanted someone to talk to post-treatment and expected their BCN would fulfil this role. In particular, Black Caribbean, Indian and Pakistani women born overseas, as well as Black Caribbean women born in the UK, wanted to talk to a BCN to obtain information. Most notably, Black Caribbean participants suggested they did not read all the written information given to them as they preferred verbal explanations alongside written information.

Women reported positive experiences of receiving information when their BCN telephoned them, as it gave them the opportunity to ask questions.

*They need to have somebody within the clinic that can go along and can go and speak to people randomly. Asking them ‘how they feel, this is what is available, have they gone to this”. Yes it’s in the* [information] *book, but not a lot of people look in the book. Because you look in the book and you’re overwhelmed by what and how much is in the book* (UK-born Black Caribbean, 51–70 years. 0–6 months post-treatment).

In addition, White British, Black African and Pakistani women born in the UK said it would be useful to talk to other women who had been through the breast cancer experience to obtain information.

Women, particularly those under 50, talked about a lack of signposting to information and support. In all but one geographical area, women haphazardly received written leaflets about life after treatment; picking up information in waiting rooms, information centres or being given information by a BCN. One Black Caribbean woman suggested a ‘*buddy’* system would be beneficial as they could point women in the direction of sources of information. Women from minority ethnic groups also wanted more signposting to people, places and sources of post-treatment information that considered their culture and religion.

*Support wasn’t there just purely for Muslim women, you know? There’s a lot of information out there for every other group, but for me personally, I couldn’t find anything.* (UK-born Pakistani, 30–50 years. 0–6 months post-treatment)

*I’ve had no information about how African Caribbean women are affected by cancer. During my research, I found out that the likelihood of Afro-Caribbean women dying of cancer is higher than other, some other races and so on. I’m not sure if that is their role [BCN], but I have had to try and find out.* (Born abroad Black Caribbean, 30–50 years. 0–6 months post-treatment)

Several women stated they felt someone should be keeping in touch with them. They would like to have been contacted by telephone to discuss how they were feeling. One older woman thought a BCN should call once a month to see how she was getting on. Instead, she felt ‘*fobbed off’* by her BCN. In contrast, women reported feeling supported if the BCN called them to see how they were.

*What I feel is missing is not having anyone. For example, I was assigned a breast care nurse and I don’t, that person hasn’t called to find out how I’m doing or how I’m getting on. We have not spoken, she has not called me to find out how am I coping…. I have not had the care that that person should have given* (Born abroad Black Caribbean, 30–50 years. 0–6 months post-treatment).

Women wanted to be guided to services to help them with the emotional impact of cancer and their wellbeing after treatment. However, they reported ad-hoc signposting to support services post-treatment. Ad-hoc signposting could be linked to availability of local support services and also the availability of someone, e.g. the BCN, to do the signposting. As a result, there was variability in the uptake of local support services. In areas where there was an established relationship between the hospital and voluntary sector services, participants reported their BCNs frequently encouraged them to access these services to aid their emotional and physical recovery. Where there was an established relationship between participants and BCNs there was greater uptake of local and national support services due to participants trusting the BCN and their recommendations.

*It would have been helpful if the hospital did give you like a list of local help, like, help service groups but there wasn’t any, there wasn’t that information* (UK-born Indian, 30–50 years. 0–6 months post-treatment).

In one locality, women across ethnic groups used a wide range of support services, including local cancer support services and/or charities. The BCNs had a close and personal relationship with women so women felt able to call them if they needed or reported being called by their BCN. As one Black Caribbean woman born in the UK put it, the BCNs have ‘*a real presence there’*. An active support group existed, supported by BCNs. In this locality, the BCN played a key role in providing support, advice and signposting to services.

*They say “we are available for you”. It’s not that you just finished your treatment and you’re off the list* (Born abroad Pakistani, 30–50 years. 0–6 months post-treatment).

There was limited uptake of post-treatment cancer services amongst Black Caribbean and Black African women, who reported being less active help seekers. Younger Black Caribbean and Black African women had work and childcare responsibilities, which they prioritised. Also, whilst some Black African women felt the option to meet other women with breast cancer should be available, they also said they would be scared or unable to use such a service. They wanted to forget about cancer and also worried that in attending support groups people would find out they had cancer, a stigmatised disease in their community.

*I’m too scared to do it* [go to a support group]. *I don’t mind, sometimes I think, well, they don’t know me, maybe I should go to where they don’t know me, but then what happens if you get there and you know somebody there?* (Born abroad Black African, 51–70 years. 0–6 months post-treatment).

## Discussion

This paper sought to explore and compare the experiences and expectations of follow-up care after treatment for breast cancer in a multi-ethnic sample of women in England. Overall, we found women’s needs and expectations were unmet under a traditional model of follow-up. As per previous research, women were left feeling unprepared for life after treatment [15, 18, 20, 22]. Many felt abandoned due to a diminution in the care and attention they had received at diagnosis and through treatment. In particular, women expected to talk to their BCN about their physical, emotional and practical concerns but were surprised and disappointed the BCN was not present at follow-up appointments and often unavailable post-treatment. As a result, women felt unsupported and did not know where to turn for advice and support.

### Survivorship care that meets women’s expectations

To mitigate some of these issues, women need to be informed about what to expect from their follow-up care. This could be achieved, in part, through an end of treatment consultation. The consultation might involve women completing a holistic needs assessment, which provides the basis for a personalised discussion with a HCP, possibly a BCN. HCP contact details and written information pertinent to the issues raised in the needs assessment would be provided, as would an individualised treatment summary [38]. This approach would appear to meet the needs of women in this study who desire a more personalised approach that acknowledges post-treatment needs go beyond the physical. Internationally, new models of survivorship care also propose women receive a written care plan on treatment completion. Like the end of treatment consultation, the care plan is based on a personalised assessment of need and thus provides tailored information, including advice on lifestyle to reduce the risk of further cancers, management of side effects, as well as signs of recurrence [39]. Signposting to relevant sources of support would also be possible.

Some of the women in this study made service suggestions similar to those being implemented internationally, for example, written care plans, end of treatment consultations and group sessions run by nurses to obtain information and support and meet other breast cancer survivors. In this respect, the new model of aftercare would appear acceptable, where traditional follow-up is not. However, women also wanted ongoing care from the consultant they saw during treatment, more scans and monitoring and for the BCN to be readily available, contrary to the changes being implemented. With around 80 % of breast cancer survivors at low risk of recurrence being risk-stratified to a self-management pathway in England [40], women’s expectations and needs might continue to be unmet under the new model of ‘aftercare’. Expectations about what ‘aftercare’ entails would need to change for women’s needs to be met.

In addition, whilst levels of satisfaction with care plans are high, a recent systematic review highlighted no significant effect on satisfaction with care, care coordination or oncological outcomes in randomised controlled trials (RCTs) [41]. However, one study included in the review suggested a positive impact on unmet needs [41]. Therefore, it may be that alternative models of care are more appropriate to meet the needs of women post-treatment. One such model is telephone follow-up. Telephone follow-up with a BCN has been evaluated as providing higher levels of satisfaction than hospital follow-up, with women reporting it was more helpful for dealing with their concerns post-treatment [42]. Women in this study wanted continuity of care from a BCN so this model of follow-up might meet their needs and expectations. Telephone follow-up is also cost-effective [43, 44] so with resources increasingly stretched, this model of care could be considered a suitable alternative to traditional follow-up.

The discussion thus far has focused on the common issues experienced by women in this study. We continue by discussing the discernible differences in experiences and expectations, particularly evident by ethnicity but also by age and SEP.

### Communication challenges

This study has demonstrated that there are cultural differences in the way HCPs and women communicate, not necessarily a difference in their post-treatment needs. Communication barriers were a key issue for women from minority ethnic groups, particularly Indian and Pakistani women born overseas who were of a lower SEP. Many women born outside the UK were unable to ask questions because they did not have the language skills, and thus confidence, to articulate their concerns. These women often relied on being accompanied to appointments, but if family members cannot attend, or women do not have someone to attend with them, there is a clear need for professional translation services to be available post-treatment. Also new models of care appear reliant on the provision of written information, which is not appropriate for women who cannot understand written English or prefer verbal communication. Written and audio (e.g. CD-ROM) translated materials in languages other than English are required.

### Culturally-tailored survivorship care

There appeared to be limited, if any, consideration of ethnic or cultural sensitivities or personalisation in follow-up appointments. This could, in part, be due to the hurried nature of appointments or the physical focus of traditional follow-up. However, it could also be due to the competency and confidence of HCPs to carry out culturally sensitive consultations. Perceptions of cancer influence emotional responses to it and the relationship between HCPs and patients [45]. It is important that HCPs elicit what those perceptions are, as well as an individual’s cultural values, and then make attempts to understand them and modify their approach in consultations to ensure the discussion is culturally appropriate to the patient [45, 46]. Betancourt et al. (2003) outlined a cultural competence framework to address ethnic disparities in healthcare, including provision of interpreter services, language-appropriate materials and HCP education on cross-cultural issues [47]. However, whether implementation of such a framework is actually possible in a short consultation is clearly debatable.

It is also important to develop tools that ensure follow-up is tailored to the individual and their specific cultural values. Indeed, it has been argued that psychosocial information, such as that contained in written care plans, should be ‘culturally and linguistically responsive’ to cultural values and social practices [48]. Consideration should also be given to ‘cultural, economic and living situation contexts’ so ‘culturally-appropriate community resources’ can be provided [48]. The testing of new models of aftercare has, so far, generally not considered contextual factors. Indeed, a review of cancer survivorship care plans highlighted that studies lack sample diversity [49] whilst another systematic review concluded that the efficacy of different models of post-treatment care need to be evaluated in a broader population of cancer survivors with differing needs and risks [50].

### Is self-management appropriate for everyone?

New survivorship services are underpinned by a self-management philosophy. The onus is often placed on survivors to look after their own health. Patient empowerment is key to the success of this model of care as it relies on survivors taking a participatory role in maintaining their health and wellbeing. A fundamental problem arises if women are unable to self-manage, as they do not have the skills, confidence or support to do so. May et al. (2014) refer to the ‘proactive’ work patients have to do to manage their illness, including complying with self-monitoring demands, self-care and coordinating care. Patients may struggle to do this as it falls alongside the demands of everyday life. If patients become overwhelmed, they may over or under utilise healthcare services [51]. Equally, patients who become overwhelmed may experience poorer health outcomes. May et al. (2014) also assert that resources to enable patients to be proactive are often not available to certain groups in society. Being ‘proactive’ requires ‘agency’ which, in part, comes through supportive social networks. It may therefore be difficult to be proactive if patients do not have these networks. ‘Agency’ can also be inhibited by poverty and co-morbidities. In addition, to be ‘proactive’, patients need to have access to services e.g. healthcare provision, which May et al. (2014) refer to as ‘opportunities’. There is unequal access to these ‘opportunities’ in society by, amongst other factors, age, ethnicity and socioeconomic status [51].

Women will need to be prepared for the fact that it is now their responsibility to be ‘proactive’ and self-manage. However, this is something some women from certain socio-demographic groups may be unable to do. For example, Indian and Pakistani women born overseas who were in a lower SEP often relied on family members to accompany them to appointments. Likewise, information had to be read to them or translated because they did not understand English. In some parts of England, survivors are now offered the opportunity to attend group ‘health and wellbeing’ clinics to obtain information and support [52]. Findings from this study suggest attendance at this type of event would be difficult or perhaps even pointless for some women born overseas who do not speak English. Attendance may also be difficult for women who do not want to attend group events because they are concerned people in their community will find out they have had cancer. Support services in this format would be inappropriate. Black African women in this study also said they would not attend group events as they had other commitments. Barriers to health-seeking previously identified include ‘competing priorities’, for example, work and family life [53]. Some women from minority ethnic groups have a tendency to ‘soldier on’ rather than seek the support they need [53]. If women do not attend events such as ‘health and wellbeing clinics’, they risk not receiving the information and support (or ‘opportunities’) [51] they require to effectively manage their own health, which could have implications for their long-term health and wellbeing. As previously discussed, personalised telephone follow-up with a BCN could be a suitable alternative to group aftercare services. It would protect the anonymity of women and arranged to fit in with their work and family commitments. Of course, any telephone service would have to be available in languages other than English to overcome potential communication barriers.

### Breast awareness post-treatment

Some Asian and Black women of varying SEP in this study reported women do not receive enough information about breast awareness to ensure they know the signs of recurrence. As new models of survivorship care rely on self-monitoring and self-referral, efforts will need to be made after treatment to ensure women are aware of signs and symptoms of recurrence. Black African and Black Caribbean women as well as older White British women report uncertainty about breast awareness, in particular, appraising non-lump symptoms [54]. In addition, knowledge about one’s own breast cancer diagnosis is generally poor in women from minority ethnic groups, which may impact care and outcomes in the future [55]. Therefore, women need to be educated as to the signs and symptoms of recurrence and given information on how to contact HCPs should they have concerns. Information specific to the individual should be included in their written care plan. Additional breast awareness materials need to be culturally sensitive and available in languages other than English. Working with communities to develop culturally appropriate materials to lessen taboos and stigma is advocated [54].

### Strengths and limitations of the study

To our knowledge, this was the first study in England to consider early cancer survivorship needs and experiences within the context of changes to follow-up in the country. There is a paucity of UK research examining the perceptions and experiences of primary breast cancer patients from diverse ethnic and social backgrounds. Adopting a qualitative research design allowed deeper exploration of the experiences of women once they finished treatment, providing a rich picture of the commonalities and differences in post-treatment experiences. The findings were based on a diverse sample, offering an insight into the differences experienced by women from a wide range of backgrounds. The study was also multi-centre in nature, providing a broader perspective on the experience of women living in different areas of England. As we found similar patient responses from all but one geographical location, which included: identifying emotional response on transition to follow-up; challenges communicating with HCPs at follow-up; and challenges finding and accessing information and support services to address unmet needs, this means we can have greater confidence in the findings, as they were not derived from just one locality.

In terms of limitations, we were only able to conduct interviews in English. As a result, no older South Asian women were able to participate in the study. The research team recognises there is a need for further research with women who are non-English speaking, but our study was unable to do so due to time and resource constraints. Therefore, a suggestion for future research would be to use interpreters or interviewers who speak additional languages to conduct interviews with women from minority ethnic groups who do not speak English. Also, our goal was to explore the experiences of women from the main ethnic groups in the UK but, as can be seen from Table 3 Bangladeshi women were missing from the sample as no one from this group was recruited to the study. The Bangladeshi population makes up one of the five main UK ethnic groups [54, 56] so are a group that should to be included in further research in this area.

Also, whilst the sample was large and multi-ethnic, it may not have been able to show us heterogeneity within the broad ethnic groupings or SEP categories due to small numbers recruited within each of these groups. In addition, we did not interview women from other minority ethnic groups that make up a growing proportion of the English population, such as Eastern European and travelling communities. Also, due to the relatively small numbers within each ethnic group, further divided by SEP, numbers were too small to conduct an in-depth analysis of the intersection between ethnicity and SEP and the impact this may have had on expectations and experience. We recognise this as a limitation of the study. However, with a larger sample, we would expect SEP to provide some explanation for the differences found between the different ethnic groups as the two are inextricably linked.

Finally, the interviewers and participants came from different ethnic backgrounds. This may have inhibited disclosure on certain topics. There is an argument that ensuring the researcher and participant are ‘matched’ on key socio-demographic criteria is helpful to the interview interaction as sharing a similar background facilitates understanding of participants’ accounts [57, 58]. Further research may benefit from utilising interviewers who come from the same backgrounds as participants. However, a disadvantage to matching is the researcher may make assumptions based on their shared background [57]. Participants might also find it helpful, or easier, to speak to someone outside their community, to ensure their diagnosis remains private.

## Conclusions

To conclude, this paper has compared the experiences and expectations of a multi-ethnic population of breast cancer survivors living in the year following treatment. As the number of cancer survivors continues to increase, Western populations become increasingly diverse and resources become ever-more stretched, the traditional model of follow-up, which focuses on detecting recurrence, is no longer a viable model of care. As we move to a new model of survivorship care, with the emphasis on self-management, women will need to be better prepared for the post-treatment period and the role they will potentially have to play in managing their symptoms and care. We do not yet know if new models of care are meeting the needs of cancer survivors, or if they are appropriate or acceptable to everyone. This study has demonstrated that there are cultural differences in the way HCPs and women communicate and access information and support, not necessarily a difference in their post-treatment needs. Therefore, further testing and potential cultural and linguistic adaptation of different models of care is necessary to ensure they are appropriate and acceptable to breast cancer survivors from different ethnic and social backgrounds, irrespective of age.

## Abbreviations

BCN, breast care nurse; HCP, healthcare professional; NS-SEC, national statistics socio-economic classification; RCT, randomised controlled trial; SEP, socioeconomic position

## Additional file

Additional file 1: Topic guide, Breast Cancer Care study. Exploring the needs of breast cancer survivors after hospital based treatment - Topic Guide. Study topic guide. (DOC 773 kb)
