# Supplementary material for: Survivorship care and support following treatment for breast cancer: a multi-ethnic comparative qualitative study of women’s experiences
Source: BMC Health Serv Res. 2016 Aug 18;16:401. doi: 10.1186/s12913-016-1625-x (PMC4989374; doi:10.1186/s12913-016-1625-x)
Supplement: Additional file 1: — Topic guide, Breast Cancer Care study. Exploring the needs of breast cancer survivors after hospital based treatment - Topic Guide. Study topic guide. (DOC 773 kb) [file 12913_2016_1625_MOESM1_ESM.doc]

**
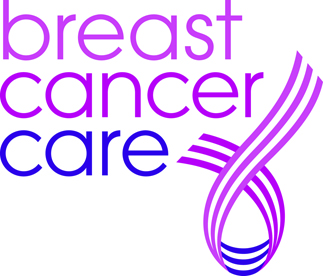
**

**Exploring the needs of breast cancer survivors after hospital based treatment**

**Topic Guide**

**Reminder of Phase 1 Aims**

To identify:

- the emotional, social and functional needs of people with breast cancer in the six month transition period following end of hospital based treatment, and examine differences that may exist between diverse population groups
- the support services breast cancer survivors are aware of/ accessing and identify what barriers/ facilitators people have experienced in accessing these services

*Interviewer note: Due to the exploratory nature of this research, it is important to encourage interview participants to discuss their views, experiences and attitudes openly. Therefore questioning should be responsive to the individual issues and experiences of each participant, following up key points raised. This topic guide lists the key themes and sub-themes to be explored at each interview, although some sections may not be relevant to some participants and so can be skipped if appropriate. The three main areas to ensure are covered include: i) physical, social and emotional well-being in transition phase; ii) areas of unmet need; iii) possible solutions to address their needs*

**The interviews will aim to:**

- Explore women’s perceptions of their emotional, social and physical/ functional well-being in this transition period, and examine potential similarities/ differences among women from different BME groups and socio-economic backgrounds;
- Identify and explore any similarities/ differences in areas of unmet needs in relation to these women’s overall health and well-being, and identify what they feel is needed to promote their well-being;
- Identify and examine any barriers and/ or facilitators that affect the women’s ability to access statutory and non-statutory information and support services post treatment;
- Examine how well their needs are currently being addressed by statutory/ non-statutory services, and the individual strategies they may have adopted to manage/ enhance their wellbeing following treatment;
- Identify and explore the best modes of providing information/support to women from different BME backgrounds or socio-economic groups in transition to survivorship, to help address their unmet needs and promote their overall well-being.

**INTRODUCTION**

*Aim: to introduce the research and set the context for the interview*

- Introduce **self and Breast Cancer Care**
- Independence from hospital and breast health team
- Non clinical role
- **Introduce research** (refer to aims above and patient information sheet):
- Funded by Big Lottery Fund
- Exploring areas where less is known – BME communities and different socio economic groups during transition
- Will inform an intervention to help people in the transition phase
- Overview details of participation (based on going through patient information sheet):
- How identified
- Explain recording, length (approx. 1 hour) and nature of discussion
- Explain outputs and reporting
- Explain confidentiality and anonymity
- Explain confidentiality and storage of data
- Explain right not to answer any questions
- Check if have any questions and are happy to continue
- Consent form

************

**SECTION 1: BACKGROUND**

*Aim: to obtain brief personal and health information linked to breast cancer*

*Before we begin to talk about your experience of life after breast cancer treatment, I would like to spend 5 minutes asking you a few short questions about yourself, where you are from etc, and a brief overview of your breast cancer diagnosis and treatment.*

Participant background

- Who you are
- Where you are from/ where you live
  - How long living in local area
  - A bit about your local community
- Who live with
  - Family responsibilities
  - Dependents

*Can you give me a brief overview about when you were first diagnosed with breast cancer?*

- How was it detected? *Who did you go and see about it?*
- How did you feel?

*And what treatment have you received?*

- How was?
- Length treatment
- When finished?

*What has been your experience so far of the follow up appointments and the aftercare you have received from the hospital since you finished treatment?*

*And what has been your experience so far of the aftercare you have received from your GP since you finished treatment?*

************

*Introduction: I am now moving on to the main part of the interview which will be divided up into two broad sections. I will firstly ask you some questions about how you have been feeling physically since completing treatment and how you are managing your day to day activities/ responsibilities. I will then move on to looking at how you are feeling in yourself, what impact breast cancer has had on you and the people closest to you, and what things you feel would help you to live your life well.*

**SECTION 2: PHYSICAL/ FUNCTIONAL NEEDS**

*Aim: to explore women’s physical and functional wellbeing following hospital treatment for primary breast cancer, identify any areas of unmet need, and factors that would help to promote physical/functional wellbeing.*

*How has your body been feeling physically since you finished treatment?*

- Is this very different to how you felt before treatment, how long been experiencing?
- Pain
- Fatigue
- Swelling/ lymphoedema
- Weight loss/ gain
- Appetite
- Sickness
- Scarring
- Cognitive
- Menopausal symptoms/ fertility
- Body image issues & self confidence in appearance; Hair loss, prosthesis)
- Sexual matters (dryness, loss libido)

*How do you feel about these physical difficulties that you are experiencing?*

- *How are these difficulties affecting your overall health and wellbeing?*
- Any other health conditions that you have (co-morbidities)
- Views and outlook for the future (e.g. fear of recurrence)
- Your feelings/ psychological wellbeing
  - Any formal diagnosis of depression/ anxiety etc/ receipt of medication
- *How are these physical difficulties/ challenges affecting your ability to carry out your day to day activities?*
- Ability to work (reminder of section 3)
- Personal care
- Household/domestic chores (cleaning, shopping, cooking)
- Childcare/ Caring responsibilities
- Hobbies
- Getting out and about (walking, gardening)

*What has helped you to cope/ manage these physical challenges?*

- *What information/ support/ advice received, sought, and who from?*

*(Facilitators/ barriers)*

- Physical strategies - e.g. Gentle exercise, rest
- Pain control/ relief - e.g. medication/ complementary therapies
- Lifestyle changes - e.g. nutritional/ dietary changes
- Personal strategies – e.g. patience, outlook
- Equipment e.g. prosthetics/ clothing
- Spiritual/ religious strategies - e.g. relaxation/ meditation, faith, prayer
- Others – e.g. GP, HCPs, BCNs, family, friends, charities, support groups

*To summarise, what would you say are the key things you need to help you feel better, and/or manage better the physical affects of life after breast cancer treatment?*

*What other information/ support would help you feel better physically/ or feel better able to manage the physical impact better?*

- From who?
- In what format?

*Going back to the day to day activities/ responsibilities you mentioned earlier, how well would you say you are managing these since completing treatment?*

- *How does this make you feel?*
- Identity/ social role
- Purpose/ Outlook
- Emotional impact/ Coping/ coming to terms with?
- *How do you think it makes those close to you feel?*

*Is there anything you feel has helped you cope/ manage the affects on these (AS IDENTIFIED BY PARTICIPANT) day to day challenges?*

- Lifestyle changes (e.g. taking on less, asking for help)
- Personal strategies – (e.g. patience, outlook)
- External support (e.g. cleaner, home help, social services)
- Others (e.g. GP, BCN, family, friends, charities, support groups)
- *What information/support/ advice received, sought, and who from?*
- (Barriers/facilitators)

*What other information/ support would help you manage your day to day activities/ responsibilities better?*

- From who?
- In what format?

************

*I would now like to talk a little bit about how you have been managing financially and with any work related issues*

**SECTION 3: FINANCE AND EMPLOYMENT**

*Aim: to explore patient financial and employment needs following hospital treatment for primary breast cancer, how these are different to pre breast cancer and what can help manage these*

*How has breast cancer impacted on you (and your family) financially?*

- Reasons for impact = Change in work status/ benefits received
- Changes/ difficulties with financial situation (e.g. Loans, Mortgage, Insurance, Pension, Paying bills)

*How has your experience of breast cancer impacted on your ability to work or your working arrangements?*

- Nature/ type
- Amount/ frequency
- Level of pay
- Any voluntary work
- Change in area of work or profession
  - Ability to do this/ barriers
- *How does this make you feel?*
- *Impact on family/friends?*

*What are your main needs now in relation to how you are currently managing your finances/ employment status?*

- *Have you received any support from others to help you to cope with any work and financial changes since finishing treatment?*
- What help/ support/ information sought to address these
  - Where did information come from
  - Any barriers to accessing this?

*What other information/support would be helpful to you around your work and financial needs following treatment/ what do you think is missing?*

IF NOT CURRENTLY WORKING:

*How do you feel about working in the future since being treated for breast cancer?*

*What are the main challenges that may influence your ability to work in the future now that you have had treatment for breast cancer?*

*What other support would you like around work and financial needs following treatment/ what do you think is missing?*

- What type of support/ help/ information and from who?
  - - Employer
    - GP
    - Employer advocate (e.g. union)
    - Hospital
    - In what format (Leaflets, Internet

************

**SECTION 4: EMOTIONAL NEEDS**

*Aim: to explore patient wellbeing and identify their emotional needs following hospital treatment for primary breast cancer, how these are different to pre breast cancer and what can help manage these*

*How have you been you feeling (emotionally) in yourself since you finished treatment?*

- Emotional impact of breast cancer treatment
- Self/ self esteem, feelings of self
- General mood and feelings since completing treatment
  - Loss self esteem
  - Isolated
  - Restless
  - Uncertain
  - Low mood
  - Depression/ anxiety (clinically diagnosed?)
  - Helpless/ worthless
  - Angry
  - Confused
  - Memory problems
  - Upset/ overly emotional
  - Fear of recurrence

*How is this different to how you felt before you had treatment?*

*How well would you say you are adjusting and coming to terms with life after breast cancer treatment?*

- Any difficulties resting/ relaxing/ sleeping
- Static or changing
  - What influences changes

*Since completing treatment, what has helped you cope/ feel better emotionally?*

- - Gentle exercise
  - Relaxation/ meditation
  - Complementary therapies
  - Rest/ sleep
  - Going out/ staying in
  - Talking to people/ people listening

Who - professionals (medics, BCNs etc) vs more informal family, friends, community, religious representative

- - - Openly or in confidence
  - Support groups (local, national, charity based, hospital based etc)
  - Self help - giving self time/ being patient with self
    - Inner strength
- **How did you hear about this?**
- **Any barriers?**

*How have these things helped you to feel better in yourself?*

*What other support would you like to be available to patients following treatment to help them adjust/ cope with the emotional impact?*

- What type of support/ help/ information?
- How? When?
  - From who?
    - GP
    - Hospital (consultant, BCN)
    - Organisations (e.g Macmillan, Breast Cancer Care)
  - In what format?
    - Leaflets
    - Internet

************

**SECTION 5: SOCIAL, CULTURAL & FAMILY**

*Aim: to explore patient social, cultural and familial needs following hospital treatment for primary breast cancer, and what can help manage these*

*What has been the impact of your diagnosis and treatment been on you and those closest to you?*

- Identity & self esteem, motivation
- Role within family, friendship groups and community
- How interact/ get on with others
- Social activities
- Things you have had to change/ stop/ give up
- Leisure pursuits
- Hobbies and recreation activities
- Time for self

*How are you coping/ managing?*

*How supported have you felt by:*

- GP, Breast Care nurse
- Your friends
- Your partner
- Your family
- Your wider community
- Any facilitators to talking/ communicating
- Nature of relationship

*How well do your family understand your needs now?*

*How well have you been able to explain to your family about how you are feeling?*

Any difficulties with this?

- Religious beliefs
- Shame/ denial
- Change in nature of relationship
  - As mother
  - As wife/ partner – loss of ‘womanhood’
- Children too young
- Don’t want to worry family

What help/ support do you think is needed for families?

*How is your diagnosis and treatment affecting your relationships?*

*Have you told others in your community about your diagnosis?*

- Why/ why not?
- How have they responded?
- How have they helped/ can they help support you?

What support/advice is needed to break down any barriers/ stigma?

*I would now like to focus a little bit more on what role your cultural and ethnic background has had/ is having on your experience of life after breast cancer.*

*Do you feel women from your community have specific/ different needs? (Why?)*

- If YES, how well do you think that these needs are understood by hospital staff?

*Would you consider yourself needing information/ support services that are specifically for women from a similar background? WHY?*

************

**SECTION 6: SPIRITUAL WELL-BEING AND NEEDS**

*Aim: to explore patients personal philosophy/ religious beliefs and identify their related needs following hospital treatment for primary breast cancer*

*How have your religious beliefs, or way you see the world changed in any way since you were diagnosed and treated for breast cancer?*

- In what way?
- Meaning/ purpose attached to diagnosis and experience of breast cancer

*Have your religious beliefs/faith/philosophy helped you in managing life following breast cancer treatment, in ways that other support or areas have not?*

*How have others sharing your faith /religious beliefs /general philosophy helped you in managing?*

*In what ways do you think this is important for health care professionals to understand the role of religion in patients lives? Why?*

************

**SECTION 7: SUPPORT AND INFORMATION SERVICES**

*Aim: to explore what services aware of, what services they have used as a source of information and support during transition phase and identify any barriers/ facilitators they have experienced in accessing these services*

*Are you aware of any information and support services for women following breast cancer in your local area? Or services that might be available nationwide?*

*What information and support services have you used to address your needs since you finished breast cancer treatment?*

(e.g. voluntary services, PALS service, support groups, courses, online groups)

- Who provided
  - Medical – GP, Consultant, Hospital, BCN
  - Family
  - Others - Social worker
- How available?

*What do you think about the support that the hospital provided you about life after breast cancer treatment?*

- Who provided?
- When? Where?
- How?
- How able felt to ask questions/ receive answers?

*What is/ was missing from this information and support?*

- How could this be addressed for you?

*What else would you liked to have known?*

*How accessible and appropriate did you find these services/ sources of information?*

- What helped access?
- What hindered?
  - Cultural understanding
  - Literacy
  - Clinician qualities e.g. approachability, time, gender

*What about the support that your family and others close to you have accessed to help them support you since you finished breast cancer treatment?*

- From where?
- When
- How useful?

************

**SECTION 8: CLOSE**

*Aim: Wrap up the interview and prepare participant for interview close*

- Check if any questions
- Overview process and write up
- Discuss if interested in being involved in focus group – complete consent form
- Give follow up information pack and £10 voucher
- Thank for time
